# Supplementary figures and images for: Neuroprotective effects of exogenous erythropoietin in Wistar rats by downregulating apoptotic factors to attenuate N-methyl-D-aspartate-mediated retinal ganglion cells death
Source: PLoS One. 2020 Apr 17;15(4):e0223208. doi: 10.1371/journal.pone.0223208 (PMC7164594; doi:10.1371/journal.pone.0223208)

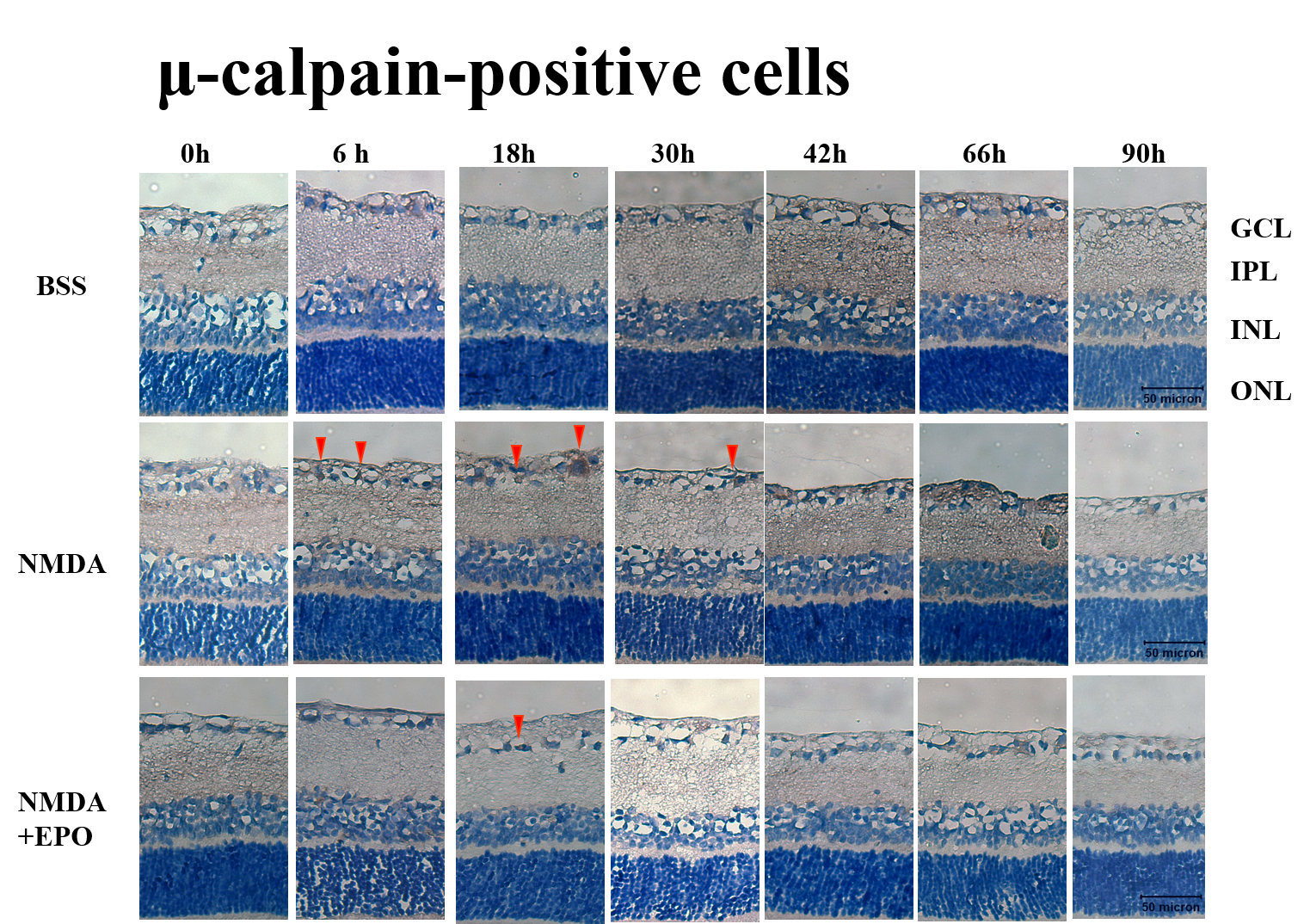

Supplement: S1 Fig — (TIFF) [file pone.0223208.s001.tiff]

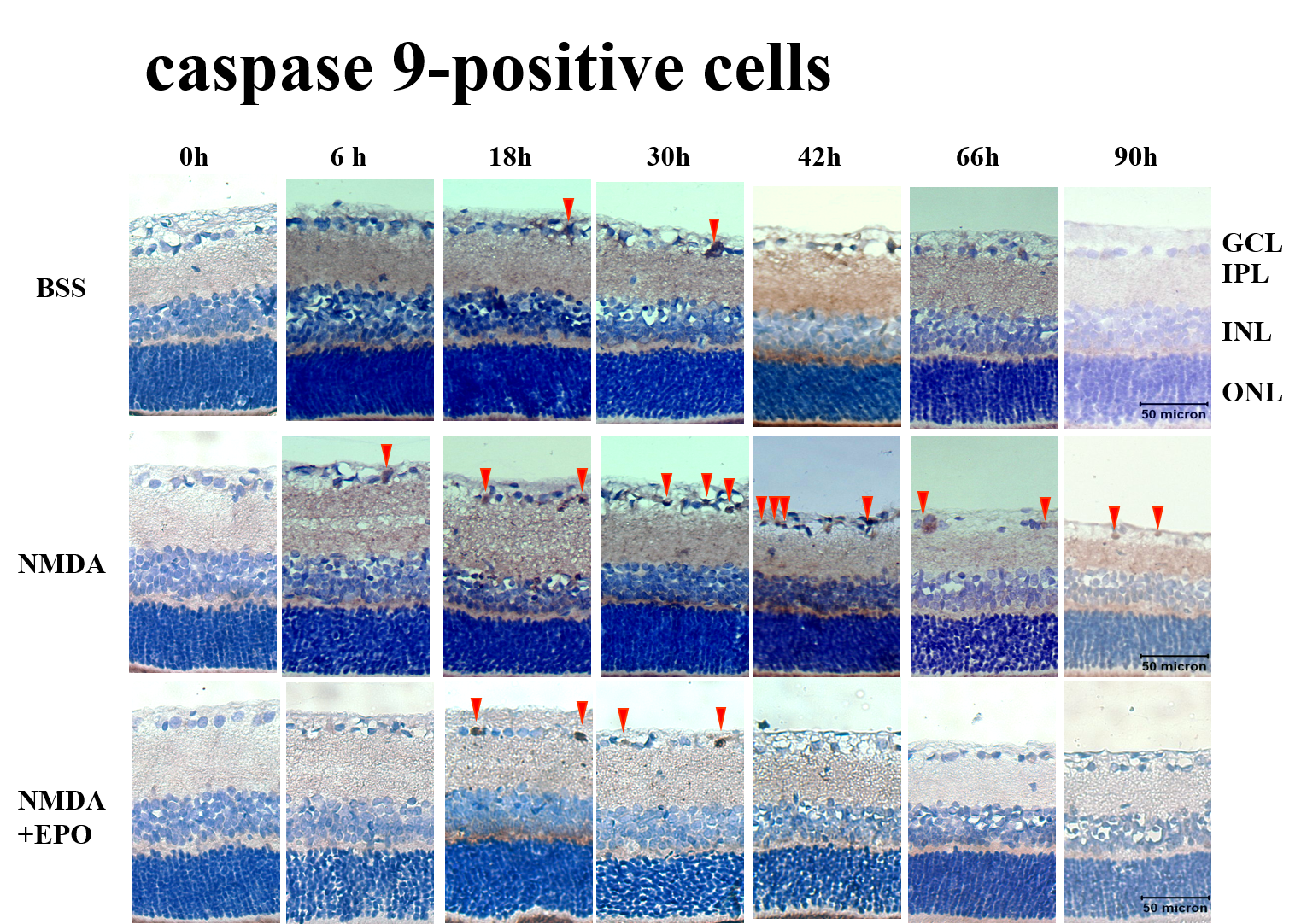

Supplement: S2 Fig — (TIFF) [file pone.0223208.s002.tiff]

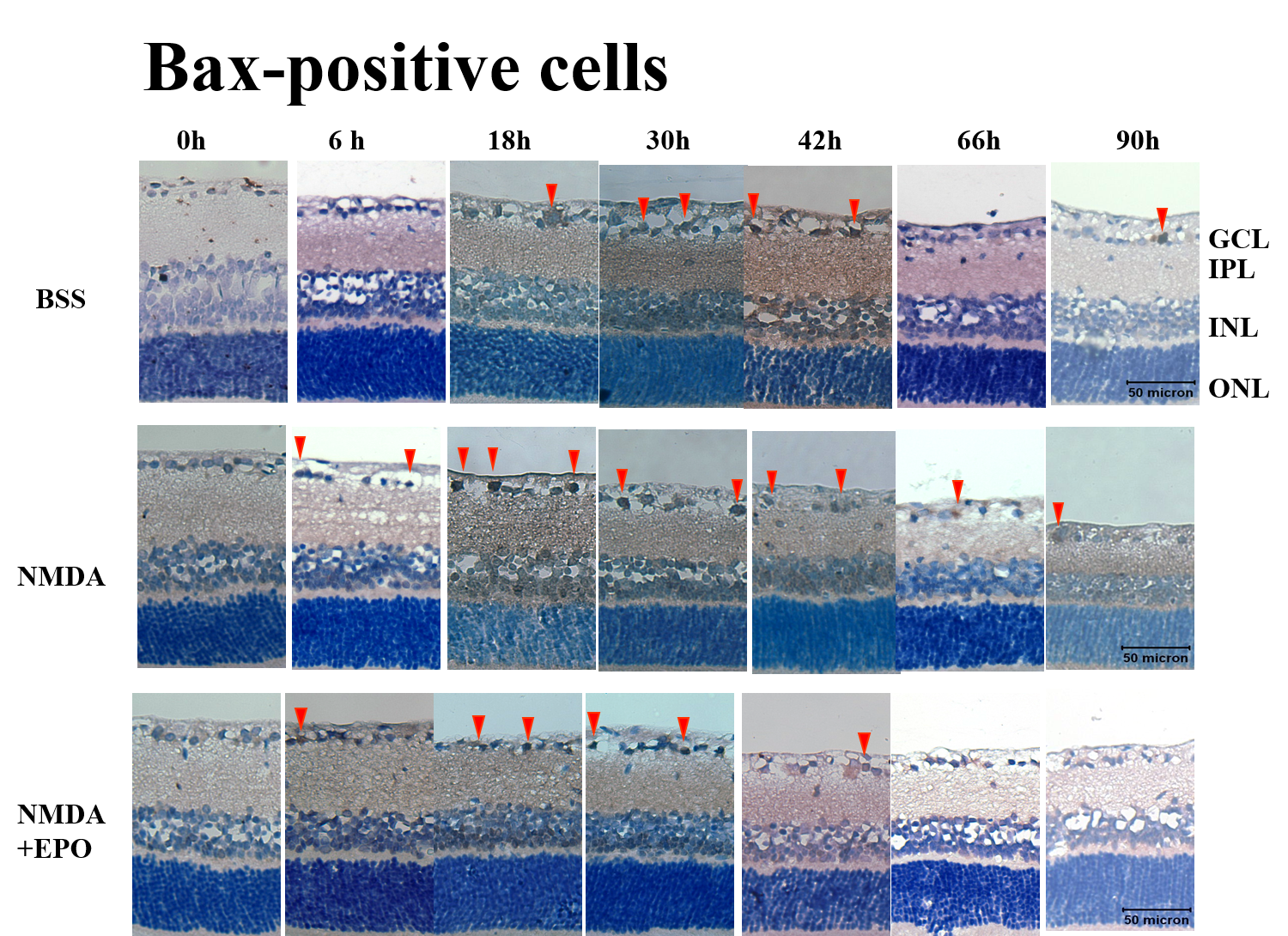

Supplement: S3 Fig — (TIFF) [file pone.0223208.s003.tiff]
